# Complementary and Alternative Drugs Use among Preoperative Patients: A Cross-Sectional Study in Italy

**DOI:** 10.1155/2012/527238

**Published:** 2011-07-21

**Authors:** Ersilia Lucenteforte, Eugenia Gallo, Alessandra Pugi, Federica Giommoni, Angelica Paoletti, Michele Vietri, Patrizia Lupi, Maristella La Torre, Gianluca Diddi, Fabio Firenzuoli, Alessandro Mugelli, Alfredo Vannacci, Francesco Lapi

**Affiliations:** ^1^Department of Preclinical and Clinical Pharmacology “M. Aiazzi Mancini”, University of Florence, Viale G. Pieraccini 6, 50139 Florence, Italy; ^2^Interuniversitary Centre of Molecular Medicine and Applied Biophysics (CIMMBA), University of Florence, Florence, Italy; ^3^Department of Labour Medicine “L. Devoto”, Section of Medical Statistics and Biometry “G. A. Maccacaro”, University of Milan, Milan, Italy; ^4^Tuscan Regional Centre of Pharmacovigilance, Florence, Italy; ^5^Anaesthesiology Unit ASL 11 Hospital, Empoli, Italy; ^6^Anaesthesiology Unit ASL 10 Hospital, Florence, Italy; ^7^Anaesthesiology Unit ASL 4 Hospital, Prato, Italy; ^8^Centre of Natural Medicine, S. Giuseppe Hospital, ASL11, Empoli, Italy; ^9^Epidemiology Unit, Regional Authority for Healthcare Services of Tuscany, Florence, Italy

## Abstract

Complementary and alternative drugs (CADs) are widely used in preoperative patients and may lead to potential interactions and adverse reactions. The aim of our study is to evaluate the prevalence and the predictors of CADs use among preoperative patients using data from an Italian survey. This cross-sectional study, which enrolled 478 patients (response rate: 83.5%), was carried out in three Tuscany hospitals (Italy). The prevalence of CADs use was 49.8%: 233 out of 238 participants used herbal products and/or dietary supplements. *Valeriana officinalis* was the most reported product (19.4%). According to univariate analysis, users were commonly identified among middle-aged or older patients; unadjusted ORs were 2.1 (95% CI: 1.3–3.3) for patients aged 48–69 years, and 3.0 (95% CI: 1.9–4.7) for those of 70–95 years, when compared with individuals aged 18–47 years. Except for education and gender, adjusted estimates showed consistent results with univariate analyses: direct association was observed with higher education, and—although not significantly—with female gender. The high prevalence of CAD use in preoperative period could be suggestive of a certain risk of adverse effects due to CADs interactions. A careful medical history of CADs consumption should be ascertained before surgery.

## 1. Introduction

The use of herbal drugs and other alternative medications is widespread across Western countries [1–3]. In Italy, 13.6% of the Italian population (about 8 million people) reported the use of nonconventional therapies, and 3.7% of herbal remedies. The latter percentage reached 4.3 in Tuscany [4]. 

Users of complementary and alternative drugs (CADs) consider these remedies safe because “natural” [5]. Nevertheless, several epidemiological studies and case reports underlined the risk of adverse events associated with their consumption, and in some clinical settings, the use of alternative medications can be particularly dangerous [6, 7]. Anaesthesia often involves the administration of drugs belonging to different classes, and patients are usually under pharmacological treatment because of their surgical condition or for other comorbidities [8]. 

Many studies have been conducted in order to evaluate the prevalence of CADs use in preoperative period [9–19]. The highest prevalence of CADs use was found in a cohort study conducted in Hong Kong (China) [19] on 601 patients undergoing surgery: 80% of them took self-prescribed traditional Chinese herbal medicines, and 8% used them within the two weeks before surgery. The lowest prevalence was reported in a survey conducted in a UK hospital setting [14] on 2723 patients: 4.8% of them took one or more herbal medications. Other surveys were conducted in USA [9–13, 16, 20], in Europe [17, 18], and Canada [15, 21]. To our knowledge, no studies on adult patients in preoperative period have been conducted in Italy.

Although there is no definite evidence, the complications that could arise from commonly used herbal medications include myocardial infarction, stroke, bleeding, inadequate oral anticoagulation, prolonged effects of anaesthesia, organ transplant rejection, and potential interactions with conventional pharmacotherapy [22]. Given these potentially serious complications, and the lack of Italian data on this topic, the prevalence and the predictors of CADs use among preoperative patients were investigated by conducting a cross-sectional survey of patients at the time of presurgical anaesthesiological visit.

## 2. Methods

This survey was conducted from November 2007 to February 2008, in three hospitals of Tuscany (Italy): Empoli, Florence, and Prato, after institutional authorization. The sample population consisted of patients admitted to the hospital in order to undergo a surgical intervention, at time of their preoperative evaluation (before surgery): thus, recall bias should be strongly reduced. All eligible participants, consecutively recruited, received written and oral information about the study, and written informed consent was obtained. 

Data were collected by means of a semistructured questionnaire administered by a trained nurse using a face-to-face interview. 

The questionnaire included socio-demographic characteristics (age, gender, education, and occupation), clinical information (chronic current diseases and the operative class risk score according to American Society of Anaesthesiologists (ASA) physical status classification), use of synthetic drugs and CADs in the latter two weeks before interview (information on name, number of medications, and the reason of use was collected). Before each interview, patients received a definition of CADs, generally defined as “any type of product manufactured from plant or with natural origin.” 

Any series of items was combined according to the methodological literature [23–27], and it was validated by an ad hoc panel of experts (pharmacologists, epidemiologists, toxicologists, pharmacists, and clinicians) of the Tuscan Regional Centre of Pharmacovigilance, a clinician of the regional referring Centre of Natural Medicine, and a group of clinicians and nurses of the three hospitals.

### 2.1. Statistical Analysis

In order to determine the predictors of CADs use, odds ratios (ORs) and the corresponding 95% confidence intervals (CIs) were estimated using univariate and multivariate logistic regression models. Each variable collected was evaluated as a predictor or protective factor whether it was related to a 10% increase or decrease of univariate OR [28]. Subsequently, it was retained in the final models according to Likelihood Ratio Test. A *P*-value below 0.05 was considered an index of statistical significance. 

Data management and statistical analysis were performed using SPSS 14.0 for Windows (SPSS Inc, Chicago, Ill) and Stata 10.0 (College Station, Tex), respectively. 

## 3. Results

Five hundred seventy-two patients were enrolled at time of their preoperative visit: ninety-five of them (16.5%) were not able to report essential information. Thus, the analysis was restricted to 478 subjects. 

Table 1 shows the characteristics of 478 enrolled patients according to selected covariates and CADs use. Patients were mainly female (57.7%), with a low-education level (33.5% with primary school degree compared to 8.4% with university one). A high percentage (33.3%) were homemakers. One-hundred seventy-nine (37.5%) patients were in the first category of ASA physical status classification, 233 (48.7%) in the second one, and 66 (13.8%) in the third category. Only one patient was in the fourth category, so he was grouped with the third level. Three-hundred patients (65%) used more than one conventional drug, and 147 (30.7%) used more than three. Overall, the prevalence of CADs use was 49.8%, and 233 out of 238 used herbal products and/or dietary supplements.

**Figure 1:**
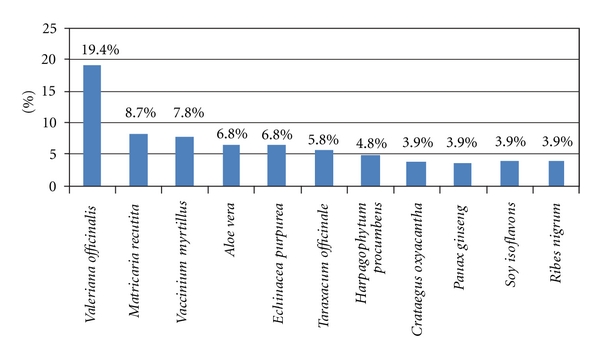
Percentages of 238 CADs users who consumed various CADs. CADs: complementary and alternative drugs.

Among 238 CADs users, drugs mainly reported were *Valeriana officinalis* (19.4%), *Matricaria recutita* (8.7%), *Vaccinium myrtillus* (7.8%), *Aloe vera* (6.8%), and *Echinacea purpurea* (6.8%). Other type of CADs used were *Taraxacum officinale* (5.8%), *Harpagophytum procumbens* (4.8%), *Crataegus oxyacantha*, *Panax ginseng*, *Soy isoflavones,* and *Ribes nigrum* (3.9%) (Figure 1). 

Table 2 gives the unadjusted ORs and the corresponding 95% CI for 238 users and 240 nonusers of CADs according to age, gender, and other selected characteristics. Advanced age was directly associated to the use of CADs: compared to patients of 18–47 years, the OR were 2.1 (95% CI: 1.3–3.3) for patients of 48–69 years and 3.0 (95% CI: 1.9–4.7) for those of 70–95 years. Direct associations were also found among retired patients compared to homemakers (OR = 3.2; 95% CI: 1.7–5.7) and for users of more than three conventional drugs when compared with nonusers (OR = 2.1; 95% CI: 1.4–3.4). With a borderline significance, the highest category of ASA physical status classification was directly related to the use (OR 1.8, 95% CI: 1.0–3.2, in patients in the third or higher category towards the lowest one). No significant associations emerged between use of CADs and gender or education.

According to Likelihood Ratio Test, mutual adjustment for age, gender, education, and occupation did not substantially change the univariate estimates, except for education: the ORs were 2.6 (95% CI: 1.4–4.7) and 3.5 (95% CI: 1.5–8.3) for subjects with high school degree and university degree, respectively (Table 3). Moreover, the use was higher in females than in males, although the association was not significant.

When homeopathic medications were excluded, the ORs did not substantially change and the predictors of use were the same observed for CADs use (data not shown).

## 4. Discussion

Our study reveals that 49.8% of 478 patients undergoing a presurgical anaesthesiological examination used an alternative medication. They were commonly identified among middle-aged and older, retired, with a high educational level, taking more than one conventional drug, and characterized by a higher ASA physical status classification. Moreover, although not significantly, the risk of CADs use was higher among females than males. 

Compared to other European studies, the prevalence of use observed in our survey was higher than those reported in a British survey including 2723 patients (4.8% used one or more herbal remedies) [14], as well as in a French multicenter study including 1057 patients (9% used one or more herbal remedies) [17]. In contrast, our estimated prevalence was lower than that reported in a northeast Scottish cross-sectional survey including 285 subjects (63% used complementary and alternative medicine) [18]. 

Compared to non-European countries, one study from China [19] reported higher prevalence, while five studies from the USA [10, 11, 13, 16, 20] and two from Canada [15, 21] reported lower prevalence. In a study carried out in USA [12], 12.9% of middle-aged and older cardiac patients confirmed vitamin use, 11.6% herbs and folk remedies use, and 3.6% use of homeopathy. In another American survey [9] of patients undergoing cardiac surgery, 53.6% used vitamins, 17.1% nutritional therapy, 9.9% herbs, and 3.0% homeopathy. Such findings variability could be explained by the definition of complementary medicine itself [29]. In fact, our survey was aimed at exploring the use of any so-called “products manufactured from herbs and/or with natural origin” and was focused on remedies that did not include, for example, acupuncture or meditation but only pharmacological-like treatment. For this reason the overall prevalence was higher or lower when compared with that reported in other investigations. In particular, our definition was less or more restrictive when compared to those European surveys which showed, respectively, a lower or a higher prevalence of CAD use [14, 17, 18].

As far as predictors of use are concerned, our results are in agreement with other studies that reported direct association between CADs use and older age [10, 11, 14, 16, 17], higher education [11], and female gender [10, 11, 14–17]. The fact that advanced age and use of concurrent conventional medications were predictors of CADs use may be due to the burden of comorbidity in older subjects [30, 31]. Indeed, although with a minor impact on the clinical condition, use of CADs with a sedative action or anti-inflammatory properties could be adopted for more common diseases and/or their symptoms among frail older patients [30]. Also, it may be likely explained by the direct association here reported between CADs use and patients with a high ASA physical status classification—as an index of more complicated patients. Moreover, patients who have a better education [32] as well as women [33] may have greater access to healthcare information. This not necessarily means, however, that women are able to correctly classify efficacy and safety of this kind of therapy [5].

Generally, in Italy, CADs do not always undergo to the same regulations as conventional medicine, and there is often little concern over purity, safety, or teratogenicity of this kind of medications [5]. Thus, from a clinical viewpoint, both herbal medications *per se* as well as their interaction with conventional drugs may cause clinical complications during surgical procedures, or the preoperative period [22, 34]. In our study, valerian (*Valeriana officinalis)* was most commonly used (19.4%). As shown in animal studies, this plant is able to induce sedation by modulating gamma aminobutyric acid neurotransmission. Theoretically, additive CNS and respiratory depression may occur when valerian is used concomitantly with opioid analgesics therefore caution is advised whenever valerian and an opiate are used in combination [35]. Additionally, valerian may increase the sedative effects of anaesthetics through a synergistic action [22]; thus it should not be combined with benzodiazepines and barbiturates [36, 37]. Accordingly, a recent study reported that animals given a combination of midazolam and valerian took significantly longer to emerge from anaesthesia compared with those treated only with midazolam [38].

Eight point seven % of subjects reported the use chamomile (*Matricaria recutita*). Chamomile contains several flavonoids, comprising apigenin, which has benzodiazepine-agonist and histamine-antagonist effects [39, 40], and tannin, which may inhibit iron absorption [34]. Interactions between *Matricaria recutita* and opioid analgesics and warfarin have been reported [35, 41]. Use of blueberry (*Vaccinium myrtillus*), aloe (*Aloe vera*), and Echinacea (*Echinacea purpurea*) were also reported. Blueberry juice contains flavonoids that may inhibit CYP enzymes, so affecting the metabolism of warfarin [42, 43]. Also ingestion of aloe can enhance the hypoglycaemic effect of glibenclamide [44], and important interactions were also reported between aloe and sevoflurane [45]. Echinacea, itself, showed some hepatotoxic potential and therefore it should not be taken together with potentially hepatotoxic drugs, in particular as a self-medication [34, 35, 46], as well as with midazolam [47].

Our study had several limitations. First, the questionnaire relied on self-reporting information; thus our results may under- or overestimate the actual prevalence of CADs use. In addition, no information on dosages was recorded. Finally, it was not possible to assess any clinical outcome. These limitations notwithstanding, our study raised several concerns about the potential risks of anaesthesiological and surgical procedures in patients exposed to CADs during the preoperative period. All the aspects here described might pose patients at risk especially when they belong to an advanced age group, and concurrent diseases and medications are present. 

In conclusion, although other studies are necessary to confirm the clinical burden of our findings, anaesthetists and surgeons should be aware of the possible complications due to the use of CADs. A careful medical history of CAD use should be taken in patients (especially older and educated patients) before surgery.

##  Conflict of Interests

The authors declare that they have no conflict of interests.

## Figures and Tables

**Table 1:** Characteristics of 478 patients at time of their preoperative visit according to general information and use of CADs.

Characteristics	No. (%)
Age (years)	
18–47	165 (34.5)
48–69	158 (33.1)
70–95	155 (32.4)
Sex	
Male	202 (42.3)
Female	276 (57.7)
Education (degree)	
Primary school	160 (33.5)
Secondary school	128 (26.8)
High school	150 (31.4)
University	40 (8.4)
Occupation	
Homemaker	159 (33.3)
Retired	81 (17.0)
Labourer	75 (15.7)
Office worker	70 (14.6)
Merchant	66 (13.8)
Others	27 (5.6)
ASA physical status classification	
1	179 (37.5)
2	233 (48.7)
≥3	66 (13.8)
Number of conventional medications	
0	171 (35.8)
1-2	160 (33.5)
≥3	147 (30.7)
Use of CADs	
No	240 (50.2)
Yes	238 (49.8)

CADs: complementary and alternative drugs.

ASA: American Society of Anaesthesiologists.

**Table 2:** Unadjusted odds ratios^a^ (ORs) and corresponding 95% confidence intervals (CI) for 238 users and 240 nonusers of CADs according to selected covariates.

Characteristics	CADs use -no. (%)		OR (95% CI)
	Yes	No	
Age (years)			
18–47	58 (24.4)	107 (44.6)	1^b^
48–69	84 (35.3)	74 (30.8)	2.1 (1.3–3.3)
70–95	96 (40.3)	59 (24.6)	3.0 (1.9–4.7)
Sex			
Male	94 (39.5)	108 (45)	1^b^
Female	144 (60.5)	132 (55)	1.2 (0.8–1.8)
Education (degree)			
Primary school	81 (34.0)	79 (32.9)	1^b^
Secondary school	51 (21.4)	77 (32.0)	0.6 (0.4–1.0)
High school	80 (33.6)	70 (29.2)	1.1 (0.7–1.7)
University	26 (10.9)	14 (5.8)	1.8 (0.9–3.7)
Occupation			
Homemaker	26 (10.9)	40 (16.7)	1^b^
Retired	107 (45.0)	52 (21.7)	3.2 (1.7–5.7)
Labourer	25 (10.5)	56 (23.3)	0.7 (0.3–1.4)
Office worker	30 (12.6)	45 (18.7)	1.0 (0.5–2.0)
Merchant	16 (6.7)	11 (4.6)	2.2 (0.9–5.6)
Others	34 (14.3)	36 (15.0)	1.5 (0.7–2.9)
ASA physical status classification			
1	76 (31.9)	103 (42.9)	1^b^
2	124 (52.1)	109 (45.4)	1.5 (1.0–2.3)
≥3	38 (16.0)	28 (11.7)	1.8 (1.0–3.2)
Number of conventional medications			
0	69 (29.0)	102 (42.5)	1^b^
1-2	82 (34.5)	78 (32.5)	1.5 (1.0–2.4)
≥3	87 (36.5)	60 (25.0)	2.1 (1.4–3.4)

^a^Estimates from logistic regression models.

^b^Reference category.

CADs: complementary and alternative drugs.

ASA: American Society of Anaesthesiologists.

**Table 3:** Adjusted odds ratios^a^ (ORs) and corresponding 95% confidence intervals (CI) for 238 users and 240 nonusers of CADs according to selected covariates.

Characteristics	OR (95% CI)
Sex	
Male	1^b^
Female	1.4 (0.9–2.1)
Education (degree)	
Primary school	1^b^
Secondary school	1.2 (0.7–2.0)
High school	2.6 (1.4–4.7)
University	3.5 (1.5–8.3)
Occupation	
Homemaker	1^b^
Retired	2.3 (1.1–4.5)
Labourer	0.9 (0.4–1.8)
Office worker	0.8 (0.4–1.8)
Merchant	2.7 (1.0–7.1)
Others	1.3 (0.6–2.8)

^a^Estimates from logistic regression models adjusted for age, gender, education, and occupation.

^b^Reference category.

CADs: complementary and alternative drugs.
